# Supplementary material for: WordCommentsAnalyzer: A windows software tool for qualitative research
Source: F1000Res. 2018 Sep 4;7:536. Originally published 2018 May 3. [Version 2] doi: 10.12688/f1000research.14819.2 (PMC6137411; doi:10.12688/f1000research.14819.2)
Supplement: Supplementary file 3 [file f1000research-7-17614-s0002.tgz › b2a79496-4ada-47c4-9682-a99ce867836d.zip!/Eight Iranian Health Journals/Journal of Health System Research _17.docx]

© Journal of Health System Research . This work is licensed under CC BY-NC 4.0 (https://creativecommons.org/licenses/by-nc/4.0/).

Volume & Issue: دوره 10، شماره 1، 1393

Title: رابطۀ بین رضایت شغلی و استرس شغلی کارکنان بهداشتی درمانی

http://hsr.mui.ac.ir/index.php/jhsr/article/view/1193

مقدمه: استرس شغلي در روابط فردي با محيط صورت مي‌گيرد كه مي‌توان سه عامل مهم آن را ويژگي‌هاي فردي شاغل، شرايط كار و برآيند آن دو دانست. استرس باعث به هم خوردن تعادل روانشناختي شده و پيامدهاي مختلفي دارد. پيامدهاي استرس شغلي منجر به نارضايتي شغلي مي‌گردد. اين مطالعه به بررسي ارتباط بين استرس شغلي و رضايت شغلي در مراكز بهداشتي درماني پرداخته است. روش‌ها: این مطالعه توصیفی تحلیلی از نوع مقطعی می‌باشد كه کارکنان تحت پوشش شبکه بهداشت و درمان خمینی شهر (مراکز، پایگاه‌ها، خانه‌ها و شبکه) در صورت داشتن حداقل 6 ماه سابقة کار، وارد مطالعه شدند. ابزار گردآوري داده‌ها پرسشنامة سه قسمتی شامل الف) اطلاعات دموگرافيك، ب) پرسشنامه استرس شغلي ديويس وهمکاران (ضريب روايي 701/0 و ضريب پايايي 863/0) و ج) پرسشنامه استاندارد توصیف شغلی اسمیت((JDI  (روایی پرسشنامه نیز 93/0) با 6 حیطه بود. پس از جمع‌آوري داده‌ها، اطلاعات از طریق نرم افزار SPSS وارد کامپیوتر شده و با استفاده از آزمون‌های همبستگی پیرسون، همبستگی اسپیرمن، t مستقل و آنالیز واریانس مورد تجزیه و تحلیل قرار گرفت، و نتایج اعلام گرديد. يافته‌ها: نتایج نشان می‌دهد که میانگین نمره رضایت شغلی 1/13± 49/44 به دست آمد. بیشترین رضایت مربوط به حیطۀ همکاران و کمترین رضایت مربوط به حقوق و مزایا می‌باشد. میانگین و انحراف معیار نمره استرس 12/18± 21/29 می‌باشد. آزمون همبستگی پیرسون نشان داد که ارتباط معنی‌دار معکوسی بین استرس شغلی و رضایت شغلی وجود دارد (001/0>P). در این مطالعه ارتباط معنی‌داری بین جنس و استرس شغلی به دست آمد، به طوری که زنان استرس شغلی بالاتری را تجربه کردند (001/0 =P ). نتیجه‌گیری: با توجه به نتایج این پژوهش، با افزایش استرس شغلی رضایت شغلی کاهش می‌یابد. از آنجایی که مراکز بهداشتی درمانی، یک سازمان با مسؤولیت مهم در جهت ارتقا سلامت جامعه هستند، پیشنهاد می‌گردد برای کاهش استرس شغلی و افزایش رضایتمندی کارکنان تدابیری اندیشیده شود.

Title: شناخت مولفه‌های تاثیرگذار بر افسردگی پس از زایمان در مادران شهر اصفهان

http://hsr.mui.ac.ir/index.php/jhsr/article/view/1156

مقدمه: افسردگي يكي از بيماري‌هاي شايع و از مشكلات خاص دوران بارداري و پس از آن مي‌باشد. افسردگی پس از زایمان سلامت مادر را به خطر انداخته و بر ارتباطات اجتماعي وي و تكامل نوزاد اثرات منفي دارد. این مطالعه با هدف بررسی شیوع افسردگی پس از زایمان و ارتباط آن با عوامل مرتبط مادران در شهر اصفهان انجام گردید. روش‌ها: این پژوهش یک مطالعه توصیفی- مقطعی است. جمعیت مورد مطالعه،  133 نفر از مادران مراجعه کننده به مراکز بهداشتی شهر اصفهان بودند که 8-4 هفته از زایمانشان گذشته بود. پرسشنامه اطلاعات دموگرافیک و مامایی و تست استاندارد بک برای سنجش میزان افسردگی به کار گرفته شد. میزان افسردگی افراد بر اساس نمره به سه دسته افسردگی خفیف، افسردگی متوسط و افسردگی شدید تقسیم شد. جهت تجزیه و تحلیل یافته از آزمون های آماری ضریب همبستگی پیرسون و رگرسیون خطی تحت نرم افزار SPSS نسخه 18 استفاده گردید. یافته‌ها: 73 نفر از مادران دارای افسردگی خفیف(10-19) و 56 نفر افسردگی متوسط (20-29) داشتند. در بین عوامل مرتبط، افسردگی پس از زایمان با تحصیلات مادر، وضعیت مالی، ناخواسته بودن بارداری، وجود سابقه سندرم پیش از قاعدگی و شغل مادر رابطه معنی‌داری وجود داشت (05/0 >P) ولی بین سایر عوامل نظیر سن مادر، جنسیت نوزاد و سابقه سقط رابطه معنی‌داری مشاهده نشد. در آناليز رگرسيون بين متغيرهاي دموگرافیک و مامایی مشخص شد كه نه متغیر در مجموع 7/27٪ واريانس افسردگی پس از زایمان را توضيح مي‌دهد. در بين اين عوامل، پيش‌گويي‌كنندگي متغيرهاي تحصیلات مادر، نوع زایمان، وضعیت مالی، ناخواسته بودن بارداری، وجود سابقه سندرم پیش از قاعدگی و شغل مادر معني‌دار بود که در اين ميان، ميزان پيش‌گويي ناخواسته بودن بارداری (24/0ß=) بيش از ساير متغيرها بود. نتیجه‌گیری: توجه به عوامل مرتبط با افسردگی پس از زایمان، تیم سلامت را در برنامه‌ریزی بهتر برای مدیریت این مشکل یاری می‌دهد. نتایج به دست آمده مطالعه حاضر، با بررسی مولفه های مرتبط با افسردگی پس از زایمان به شناخت برخی عوامل تاثیرگذار بر افسردگی بعد از زایمان کمک می‌کند تا مادران در فرایند زایمان، کمترین دغدغه روحی ور وانی را متحمل گردند.

Title: رابطه بين مولفه هاي هوش هيجاني کارکنان دانشگاه علوم پزشکي اصفهان با ابعادعدالت سازماني

http://hsr.mui.ac.ir/index.php/jhsr/article/view/1341

مقدمه: امروزه مسئله عدالت از جمله مهم ترين مفاهيمي است که بر بقا و ادامه حيات سازمان ها تاثير گذار است؛ چراکه ادراک عدالت سازمان از جمله الزامات هر نوع مشارکت اجتماعی است. پژوهش حاضر با هدف بررسي رابطه بين مولفه هاي هوش هيجاني کارکنان دانشگاه علوم پزشکي اصفهان با ابعادعدالت سازماني ایشان انجام گرفته است. روش‌ها: اين تحقيق توصيفي از نوع همبستگي مي‌باشد و در سال 1391انجام گرفته است.جامعه پژوهش کارکنان ستاد دانشگاه علوم پزشکي اصفهان با تعداد872 نفر بوده است .داده‌ها با استفاده از پرسشنامه‌هاي عدالت سازمانيColquitt، پرسشنامه هوش هيجانيPetrides و Furnham جمع‌آوري گرديد. براي تحليل داده‌ها از شاخص‌هاي توصيفي، پراکندگي و تمايل مرکزي استفاده شد و از سوي ديگر جهت تحليل داده‌ها از روش‌هاي همبستگيPearson، تحليل رگرسيون و تحليل واريانس با استفاده از نسخه 16 نرم افزار Amos  استفاده شد. يافته‌ها: از نظر جامعه آماري مورد پژوهش، رابطه بين هوش هيجاني و عدالت توزيعي، با292/0= r و رابطه بين عدالت رويه‌ای و هوش هيجاني، با 297/0= r و رابطه بين عدالت اطلاعاتي و هوش هيجاني، با616/0= r در سطح اطمینان95% معني‌دار است. لیکن شواهد حاکی از آن است که رابطه بين هوش هيجاني و عدالت بين فردي با 095 /0= r معنی‌دار نمی‌باشد. نتيجه‌گيري: بین عدالت رویه‌ای، عدالت اطلاعاتی و عدالت توزیعی با مولفه‌های هوش هیجانی رابطه معنی‌دار وجود دارد و با عنایت به این نقاط مثبت موجود در بین کارکنان دانشگاه می‌توان شاخص‌های ارزشمند موجود در مولفه‌های هوش هیجانی را در بین ایشان نهادینه کرد .

Title: رابطه مؤلفه‌هاي تعهد سازماني با عوامل مدل پستل از ديدگاه مشمولين قانون خدمت پزشكان و پيراپزشكان دانشگاه علوم پزشکی اصفهان (رویکردی استراتژیک)

http://hsr.mui.ac.ir/index.php/jhsr/article/view/1346

مقدمه: مفهوم تعهد سازماني توجه بسياري از محققان را به خود جلب کرده است، چراکه تعهد کارکنان يک نيروي قوي و موثر در موفقيت سازمان‌ها محسوب مي‌شود. پژوهش حاضر با هدف بررسي رابطه مؤلفه‌هاي تعهد سازماني با عوامل منتخب مدل پستل از ديدگاه مشمولين قانون خدمت پزشكان و پيراپزشكان دانشگاه علوم پزشکی اصفهان انجام گرفته است. روش‌ها: اين تحقيق توصيفي از نوع همبستگي است و در سال 1391انجام گرفته است. جامعه پژوهش کليه پرسنل مشمول قانون خدمت پزشكان و پيراپزشكان شاغل در مراکز بهداشتی و درمانی دانشگاه علوم پزشکي اصفهان با تعداد 1612 نفر بوده است. داده‌ها با استفاده از پرسشنامه محقق ساخته عوامل منتخب مدل پستل از نظر صاحبنظران منابع انسانی دانشگاه وپرسشنامه بومی شده تعهد سازماني  Allenو Meyer (1997) جمع‌آوري گرديد. براي تحليل داده‌ها از شاخص‌هاي توصيفي، پراکندگي و تمايل مرکزي استفاده شد و از سوي ديگر جهت تحليل داده‌ها از روش‌هاي همبستگي Pearson وتحليل واريانس با استفاده از نسخه 16 نرم‌افزارSPSS  استفاده شد. يافته‌ها: نتايج پژوهش نشان داد که ضريب همبستگي بين تعهد عاطفي با عوامل اقتصادي، اجتماعي و قانوني، ضريب همبستگي بين تعهد عقلایي با عامل قانوني و همچنين ضريب همبستگي بين تعهد هنجاري با عوامل اقتصادي و اجتماعي در سطح 05/≥p معني‌دار است. نتيجه‌گيري: تلاش در جهت ارتقاي توانمندسازي کارکنان يک استراتژي ارزشمند براي بهبود تعهد سازماني ايشان است و لازم است که مديران ارشد سازماني با مهيا کردن شرايط مناسب در جهت ارتقاي هر چه بيشتر تعهد سازماني پرسنل خود گام بردارند.

Title: بررسي وضعيت افسردگي و اضطراب در بين مبتلایان به سرطان پستان مراجعه کننده به بیمارستان تخصصی سرطان اصفهان

http://hsr.mui.ac.ir/index.php/jhsr/article/view/1401

مقدمه: مطالعات حاکی از شیوع قابل توجه و آمار بالای اضطراب و افسردگی در مبتلایان به سرطان پستان در مقایسه با سایر انواع سرطان‏هاست. با توجه به اینکه مولفه‌های اضطراب و افسردگی، دیگر شاخص‌ها و پارامترهای مهم همچون وضعيت روانشناختي بیمار، كيفيت زندگي بيماران، سیستم ایمنی بیمار و سير بيماري، بازده درمان، مدت بستري و حتي طول عمر بيمار را تحت تاثیر قرار می‏دهد، لذا محققین بر آن شدند تا مطالعه‏ای را با هدف بررسی وضعیت اضطراب و افسردگي در این بیماران ، طراحی و اجرا نمایند. روش‏ها: مطالعه حاضر از نوع توصیفی تحلیلی بود. 297 نفر از مبتلایان به سرطان‌پستان مراجعه‌کننده به مرکز درمانی تخصصی سرطان در سال 1390 به روش نمونه‌گیری آسان وارد‌ مطالعه‌گردیدند. و ابزار گردآوری اطلاعات، پرسشنامه استاندارد سنجش اضطراب و افسردگی DASS-42 بود. داده‏ها با روش‏های آمار توصیفی و استنباطی با استفاده ‌از SPSS نسخه 18و حدود اطمینان 95٪ مورد تجزیه و تحلیل قرارگرفتند. یافته‏ها: بر اساس یافته‏های پژوهش میزان شیوع افسردگی در مبتلایان به سرطان پستان 5/34٪ و اضطراب 24٪ بود. اکثریت نمونه‏ها دارای افسردگی و اضطراب متوسـط به ترتیب با شیوع 6/33 و 9/66 ٪ بودند. نتایج حاکی از آن بود که بین سطح تحصیلات، وضعیت تأهل، اشتغال، نوع درمان و مدت زمان سپری شده از تشخیص با اضطراب ارتباط آماری معنی‏داری وجود نداشت؛ همچنین  بین افسردگی نمونه‏های پژوهش با سن، جنس، وضعیت اشتغال، مدت زمان سپری شده از تشخیص و نوع درمان آنان ارتباط آماری معنی‏داری وجود نداشت (05/0P> )، لیکن بر اساس یافته‏های پژوهش بین تعداد فرزندان و اضطراب نمونه‏های پژوهش (001/0P<)؛ همچنین بین افسردگی مبتلایان به سرطان پستان با سطح تحصیلات (03/0=P )، وضعیت تأهل (05/0=P ) و تعداد فرزندان (02/0=P) ارتباط معنی‏دار آماری وجود داشت. نتیجه‏گیری: نتایج مطالعه حاضر نشان داد که اضطراب و افسردگی از جمله اختلالات‏روانی شایع در مبتلایان به سرطان پستان در کشور است. با توجه با تأثیرات قابل ملاحظه اضطراب و افسردگی در زمینه‏های مختلف زندگی فردی و اجتماعی، سلامت‏روانی و جسمانی، درمان و پیشرفت بیماری، پرداختن به مبحث افسردگی و اضطراب از اهمیت و لزوم خاصي برخوردار است و لزوم توجه بیشتر مسؤولین بهداشتی – درمانی را به این مقوله طلب می‏کند.

Title: ارزیابی فرهنگ ارائه‌دهندگان خدمات درمانی نسبت به ایمنی بیمار در میان کارکنان بیمارستان‌های شهر تهران

http://hsr.mui.ac.ir/index.php/jhsr/article/view/1331

مقدمه: بهبود ایمنی بیمار یک اولویت بین‌المللی مشترک است چرا که خطاهای بسیار زیاد در حال حاضر در اعمال مراقبتی و درمانی بیمار در حال روی دادن هست که در نتیجه میلیون‌ها نفر ممکن است از صدمات پزشکی حاصل از خطاهای پزشکی قابل اجتناب جان خود را از دست بدهند. در این مسیر این امر مشخص شده که فرهنگ ایمنی یک فاکتور اساسی موثر بر ایمنی بیمار است. هدف از این مطالعه ارزیابی فرهنگ ارایه‌دهندگان خدمات درمانی نسبت به ایمنی بیمار در میان کارکنان بیمارستان های شهر تهران می‌باشد. روش‌ها: این مطالعه توصيفي مقطعي در سال 1389 بر روي 348 نفر از پرسنل درمانی بیمارستان‌های شهر تهران انجام گرفت. آزمودني‌ها به روش نمونه‌گيري تصادفی ساده انتخاب شده و پرسشنامه‌اي كه شامل سه بخش كلي اطلاعات دموگرافيك، پرسشنامه فرهنگ ایمنی و ادراک کارکنان نسبت به ایمنی بیماران بود به صورت خودگزارش دهی از شرکت‌کنندگان تكميل گرديد. داده‌هاي جمع‌آوري شده با نرم‌افزار SPSS نسخه 16تجزیه و تحلیل گرديد. یافته‌ها: یافته‌های مطالعه حاضر نشان داد که ادراک کارکنان نسبت به فرهنگ ایمنی بیمار در بیمارستان‌ها به صورت متوسط می‌باشد. بین نوع بیمارستان، سن، درجه تحصیلات، شغل، سابقه کاری با فرهنگ ایمنی بیمار رابطه معنی‌داری وجود داشت (05/0>P). نتیجه‌گیری: فرهنگ ایمنی بیمار در بیمارستان‌ها به صورت متوسط می‌باشد. با توجه به این نتایج مداخلات لازم جهت ارتقا سطح فرهنگ ایمنی در این بیمارستان ضروری به نظر می‌رسد.

Title: بررسی نقش حمایت عاطفی در کیفیت زندگی سالمندان شهرکرد

http://hsr.mui.ac.ir/index.php/jhsr/article/view/1244

مقدمه: حمايت اجتماعي يك شبكة اجتماعي است كه براي افراد منابع روان شناختي و محسوسي را فراهم مي كند تا بتوانند با شرايط استرس زاي زندگي و مشكلات روزانه كنار بيايند. هدف از این پژوهش تعیین میانگین نمره ارتباط حمایت اجتماعی (بعدعاطفی) و کیفیت زندگی سالمندان  می باشد. روش‌ها: این مطالعه  توصیفی و جامعه آماری 356 نفر  از سالمندان ساکن شهرکرد بود. برای گردآوری اطلاعات از پرسشنامه‌های استاندارد (حمایت اجتماعی نوربک و سنجش کیفیت زندگی سالمندان لیپاد استفاده شد. تعداد سؤالات استفاده شده در این مطالعه شامل 5 سؤال دموگرافیک، 29 سؤال کیفیت زندگی و 7 سؤال حمایت اجتماعی بود. نحوه نمره‌گذاری برخی از پرسش‌ها به صورت بسیار خوب، خوب، بد، بسیار بد، برخی به صورت بدون سختی، با سختی، فقط با کمک، هیچ نمی‌توانم، تعدادی به صورت هیچ وقت، به ندرت، اکثر اوقات، همیشه، تعدادی به صورت بلی، کاملاً می‌توانم، بیشتر کارها را می‌توانم، تنها بعضی کارها را می‌توانم، هیچ نمی‌توانم، برخی به صورت تقریباً همیشه، اکثر اوقات، به ندرت، هیچ وقت، برخی به صورت بسیار راضی، راضی، ناراضی، بسیار ناراضی و برخی به صورت بسیار به ندرت، به ندرت، اکثر اوقات و همیشه بود که با توجه به نوع آنها از 5ـ 1 نمره‌گذاری شد. پرسشنامه‌ها با رعایت ملاحظات اخلاقی و محرمانه بودن پاسخ‌ها توزیع و بررسی گردید. در نهایت داده‌ها با استفاده از نرم‌افزار SPSS و آنالیزهای آمار توصیفی و آزمون همبستگی پیرسون تجزیه و تحلیل گردید. یافته‌ها: یافته‌ها نشان داد که زنان سالمند در مقایسه با مردان سالمند از کیفیت زندگی و حمایت عاطفی  پایین تری برخوردار بودند. همچنین ضریب همبستگی پیرسون نشان داد که رابطه معنی‌داری بین حمایت اجتماعی (بعد عاطفی ) و کیفیت زندگی سالمندان وجود دارد (54/.= r، 05/0= P) نتیجه‌گیری:با توجه به رابطه معنادار بین حمایت اجتماعی عاطفی و کیفیت زندگی به نظر می‌رسد که توجه به نقش حمایت اجتماعی (بعد عاطفی) در بهبود کیفیت زندگی سالمندان می‌تواند نتایج سودمندی را به دنبال داشته باشد. بنابراین حمایت‌های اجتماعی خصوصاً حمایت عاطفی به عنوان منبعی ارزان در جهت ارتقای سلامتی و در نتیجه بهبود کیفیت زندگی سالمندان مطرح می‌باشد و ضرورت دارد برنامه‌ریزان زمینه برخورداری افراد سالمند را از این حمایت ها فراهم نمایند.

Title: بررسی عوامل اجتماعی-جمعیتی مؤثر بر میزان باروری در شهرستان شهرضا

http://hsr.mui.ac.ir/index.php/jhsr/article/view/1140

مقدمه: باروری به عنوان یکی از عوامل تأثیرگذار بر رشد جمعیت، تحت تأثیر عوامل مختلف قرار دارد. بنابراین شناسایی عواملی که می‏تواند این شاخص را متأثر سازد از اهمیت خاصی برخوردار است. هدف این مطالعه بررسی مقايسه‏ای ميزان باروري و عوامل اجتماعي- جمعيتي مؤثر بر آن بین زنان 15-49 ساله تحت پوشش سازمان بهزيستي و کميته امداد امام (ره) با زنان غير تحت پوشش در شهرستان شهرضا بود. روش‏ها: روش به کار رفته در این پژوهش روش پیمایش بود. جامعه آماری این تحقیق را کلیه زنان همسردار 49-15 ساله تحت پوشش سازمان بهزیستی و کمیته امداد امام (ره) و زنان همسردار 49-15 ساله غیر تحت پوشش، شهرستان شهرضا تشکیل می‏دهند، که 418 نفر به عنوان نمونه، تعيين و با استفاده از روش نمونه‏گیری تصادفی چند مرحله‌ای انتخاب شدند. ابزار گردآوری اطلاعات، پرسشنامه محقق ساخته شامل 8 سؤال بود. جهت تجزیه و تحلیل داده‏ها از آمار توصیفی (فراوانی، درصد و میانگین) و آمار استنباطی (ضريب همبستگي پيرسون، تي مستقل، تحليل واریانس یک ‏طرفه) در سطح معنی‌داری کمتر از 01/0، با كاربرد نرم‌افزار SPSS نسخه 16 استفاده گرديد. یافته‏ها: نتایج به دست آمده نشان ‏داد، بین متغیرهای وضع تحصیلات، محل سکونت، طول مدت ازدواج، سن اولین ازدواج، ترجیح جنسی، تعداد سقط جنین (گروه تحت پوشش)، با میزان باروری در سطح معنی‌داری کمتر از 01/0 رابطه معنی‌داری وجود داشت. در حالی که، در میان متغیرهای بررسی شده بین متغیرهای محل سکونت، ترجیح جنسی و تعداد سقط جنین (گروه غیر تحت پوشش)، با میزان باروری در سطح معناداری کمتر از 01/0 رابطه معناداری وجود نداشت. نتیجه‏گیری: با توجه به نتایج تحقیق، ارتقاء سطح سواد زن، فرهنگ‏سازی در زمینه‏ی نگرش مثبت به داشتن فرزند دختر، فراهم ساختن امکانات لازم و برگزاری کلاس‏ها در جهت آگاه‏سازی روستانشینان از روش‏های جلوگیری از بارداری می‏تواند در جهت کاهش باروری مؤثر باشد.

Title: بررسی اپیدمیولوژیک کلستاز نوزادی در بیمارستان شهید صدوقی یزد: یک مطالعه گذشته نگر شش ساله

http://hsr.mui.ac.ir/index.php/jhsr/article/view/1374

مقدمه: كلستاز نوزادي از بیماری‌های مهم كبدي و مجاري صفراوي نوزادن است و گستره وﺳﻴﻌﻲ از وﺿﻌﻴﺖﻫﺎيﻛﻠﻴﻨﻴﻜﻲ ﺷﺎﻣﻞ هپاتيت ايديوپاتيك نوزاد، آترزي مجاري صفراوي خارج كبدي،كيست كلدوك و ﻋﻮاﻣﻞ ﻋﻔﻮﻧﻲ را شامل می‌شود.عدم تشخيص به موقع این بیماری می‌تواند عوارض غيرقابل جبراني در پی داشته باشد. ﻣﻄﺎﻟﻌﻪحاضر با ﻫﺪف تعیین علل کلستاز نوزادی به روش ﻫﻴﺴﺘﻮ ﭘﺎﺗﻮﻟﻮژﻳﻚ در بیمارستان شهید صدوقی یزد طی سال‌های 1385 تا 1390 انجام گرفت. روش‌ها:اين پژوهش يك مطالعه گذشته‌نگر بود و به صورت سرشماری در میان 52 پرونده بایگانی موجود در بخش پاتولوژی مربوط به نوزادان مبتلا به کلستاز نوزادی بستری شده در بیمارستان شهید صدوقی یزد در طی سال‌های 1385 تا 1390 انجام گرفت. جهت جمع‌آوری داده‌ها جمعیت‌شناختی، بالینی و آسیب شناختی از چک لیست استفاده شد. داده‌های جمع‌آوری شده توسط نرم‌افزار آماری SPSS نسخه 18 با بهره‌گیری از آمار توصیفی و آزمون تحلیلی کای دو در سطح معنی‌داری 05/0 مورد تجزیه و تحلیل قرار گرفتند. یافته‌ها:4/40٪ دختر و 6/59 ٪ پسر بودند. به ترتیب فراوانی هپاتیت نوزادی و آترزی مجاری صفراوی خارج کبدی (4/90٪) بیش از سایر علل بود. مبتلایان به آترزی مجاری صفراوی خارج کبدی سن بیشتری داشتند. پسرها بیش از دختران به آترزی مجاری صفراوی خارج کبدی مبتلا بودند. هپاتومگالی در آترزی مجاری صفراوی خارج کبدی بیشتر از هپاتیت نوزادی بود. نوزادان مبتلا به هپاتیت نوزادی در مقایسه با نوزادان مبتلا به آترزی مجاری صفراوی خارج کبدی بیشتر دارای علامت مدفوع روشن بودند (017/0P=). نتیجه گیری: هپاتیت نوزادی و آترزی مجاری صفراوی خارج کبدی مهم‌ترین علل کلستاز نوزادی بودند. نشانه‌های بالینی عوامل تشخیص افتراقی مناسب میان آترزی مجاری صفراوی خارج کبدی و هپاتیت نوزادی نیستند. پیشنهاد می‌شود از تست بیوپسی کبد استفاده شود.

Title: مقایسه سه پرسشنامه سنجش کیفیت زندگی بیماران نارسایی قلبی در برنامه بازتوانی قلبی

http://hsr.mui.ac.ir/index.php/jhsr/article/view/1391

مقدمه :بیماری نارسایی قلبی یک بیماری مزمن است که تاثیرات منفی بر کیفیت زندگی افراد می‌گذارد. یکی از اهداف مهم درمان بیماری نارسایی قلبی علاوه بر پیشگیری از پیشرفت بیماری، بهبود کیفیت زندگی بیماران است. هدف از این مطالعه مقایسه سه پرسشنامه کیفیت زندگی بیماران نارسایی قلبی با تمرکز بر دو پرسشنامه اختصاصی بیماران قلبی که در برنامه بازتوانی قلبی استفاده می شوند، می‌باشد. روش‌ها: سه پرسشنامه سنجش کیفیت زندگی، شامل فرم کوتاه 36 سؤال و دو پرسشنامه اختصاصی شامل پرسشنامه کیفیت زندگی در بیماران آنفارکتوس MacNew) ) و پرسشنامه کیفیت زندگی مینه سوتا (MLHF) در 60 بیمار نارسایی قلبی شرکت‌کننده در برنامه بازتوانی قبل از بازتوانی و سه ماه پس از اتمام بازتوانی تکمیل شد. پرسشنامه سنجش خصوصیات زمینه‌ای جمعیتی نیز در بیماران تکمیل شد. ضریب همبستگی پیرسون در قبل و بعد از بازتوانی بین ابعاد پرسشنامه‌ها محاسبه شد. مقدار تغییرات هر بعد از دو پرسشنامه‌ها نیز با یکدیگر مقایسه شد. پس از آنالیز پرسشنامه‌ها طبق دستورالمعل سازنده آنها، داده‌ها وارد نرم افزار SPSS نسخه 18 شد. نمره کیفیت زندگی بیماران در دو پرسشنامه اختصاصی بیماران بر حسب طبقه‌بندی NYHA قبل از انجام بازتوانی محاسبه شدند و از ضریب همبستگی پیرسون و t-test برای تجزیه و تحلیل داده‌ها استفاده شد. یافته‌ها: ضریب همبستگی پیرسون بین دو پرسشنامه اختصاصی قلبی با SF-36 در سه ماهه پس آزمون در ابعاد مشابه (اعتبار همگرایی) معنی‌دار بود (05/0 p-value≤). ضریب همبستگی پیرسون در بعد کلی پرسشنامه MacNew در پیش از آزمون با تمام ابعاد پرسشنامه MLHF معنی‌دار بود. علاوه بر این ضریب همبستگی پیرسون در تمام ابعاد MacNew با تمام ابعاد MLHF در مقطع سه ماهه پس آزمون معنی‌دار شد. تغییرات بعد کلی و روانی پرسشنامه MacNew با تغییرات دو بعد فیزیکی و کلی پرسشنامه MLHF همبستگی معنی‌داری رانشان داد. ضریب آلفای کرونباخ در پرسشنامه MLHF طیفی از 64/0 تا 76/0 را شامل می‌شد و برای پرسشنامه MacNew طیفی از 71/0 تا 93/0 محاسبه شد. میانگین نمره کیفیت زندگی بیماران بین دو طبقه NYHA در دو  بعد روانی و بعد کلی پرسشنامه MLHF معنی‌دار بود، اما در هیچ یک از ابعاد پرسشنامه MacNew معنی‌دار نبود. نتیجه‌گیری: ضریب همبستگی پیرسون در ابعاد فیزیکی و روانی سه پرسشنامه مثبت و معنی­دار بود با توجه به نتایج مطالعه می‌توان نتیجه گرفت که پرسشنامه MLHF برای بیماران نارسایی قلبی از حساسیت بیشتری نسبت به تغییرات شدت بیماری برخوردار است. مطالعات بیشتری در زمینه تعیین حساسیت دو پرسشنامه بر اساس معیارهای دیگر مانند خود گزارش‌دهی بیمار از سلامت خود، افسردگی بیمار و اضطراب بیمار لازم است.

Title: بررسی موانع ا نجام پژوهش از دیدگاه اعضای هیأت علمی دانشگاه علوم پزشکی اصفهان

http://hsr.mui.ac.ir/index.php/jhsr/article/view/1176

مقدمه: دانشگاه‌ها مهم‌ترين محل توليد علم محسوب شده و سامان‌دهي به تحقيقات دانشگاهي از مهم‌ترين راهبردهاي مؤثر در پيشرفت جامعه است پژوهش حاضر به منظور تعیین موانع انجام پژوهش از دیدگاه اعضاي هیأت علمی دانشگاه علوم پزشکی اصفهان انجام گرفت. روش‌ها: این مطالعه توصیفی مقطعی به منظور تعیین موانع پژوهش از دیدگاه اعضا هیأت علمی دانشگاه علوم پزشکی اصفهان انجام گرفت. نمونه پژوهش شامل تمامی اعضا هیأت علمی شاغل در دانشگاه علوم پزشکی اصفهان بودند. داده‌ها به وسیله پرسشنامه‌ای که روایی و پایایی آن مورد تایید قرار گرفت جمع‌آوری شد. پرسشنامه‌ها از طریق پست به تمام اعضای هیات علمی ارسال گردید و در نهایت تعداد121 پرسشنامه توسط اعضای هیأت علمی تکمیل گردید. پس از وارد کردن اطلاعات در نرم‌افزار آماري  SPSSنسخه 16 با آزمون‌های آماری توصیفی مورد تجزیه و تحلیل قرار گرفت. یافته‌ها: در مجموع 121 پرسنامه عودت داده شد. از مجموعه افراد شرکت‌کننده در پژوهش 4/26٪ زن (32نفر) و 6/73٪ (89 نفر) مرد و مدرک تحصیلی  6/30٪ دکترای تخصصی(PhD)  بود. مهمترین موانع پژوهش کمبود وقت و مشغله کاری زیاد، مقررات دست و پا گیر اداری، عدم استفاده بهینه از نتایج پژوهش و روند طولانی تصویب طرح‌های تحقیقاتی به دست آمد. نتیجه‌گیری: از ديدگاه اعضای هيأت علمي موانع متعددي فعاليت‌هاي تحقيقاتي تحت تأثير قرار می‌دهد. بازنگري در فرايند تصويب طرح‌هاي پژوهشي، توانمندسازي نيروي انساني، توجه به كاربست نتايج، طراحي سيستم اطلاعاتي و ارتباطاتي نيز مي‌تواند در رفع مشكلات و موانع پژوهش مؤثر باشد.

Title: حمایت اجتماعی و عوامل موثر بر آن‌ در مهاجران افغان شهرستان ساوجبلاغ

http://hsr.mui.ac.ir/index.php/jhsr/article/view/1061

مقدمه: مهاجران خارجی بخشی از جمعیت هر کشوری را تشکیل می‌دهند. در ميان جمعيت مهاجر خارجي در ايران سهم مهاجران و اتباع كشور افغانستان بسيار قابل توجه است. یکی از عوامل تأثیرگذار بر روی سلامت جسمانی و روانی این افراد، حمایت اجتماعی می‌باشد. این مطالعه با هدف بررسي وضعيت حمایت اجتماعی و عوامل موثر بر آن در مهاجرين افغان انجام شد. روش‌ها: این مطالعه به صورت مقطعی در سال 1391 و بر روی 400 نفر از مهاجران افغان شهرستان ساوجبلاغ انجام گرفت. برای جمع آوری داده‌های پژوهش از پرسشنامه‌های جمعیت شناختی و حمایت اجتماعی وکس استفاده شد. تجزیه و تحلیل داده‌ها با انجام آمار توصیفی و آزمون‌های همبستگی، کروسکال والیس و من ویتنی به کمک نرم افزار SPSS نسخه 18 صورت گرفت. سطح معني‌داري کمتر از 05/0 در نظر گرفته شده است (05/0P<). یافته‌ها: نمونه‌های مطالعه شامل 231 نفر مرد (7/57%) و 169 نفر زن (3/42%) بودند. ميانگين کلی سن مهاجران 54/8±01/26، میانگین سنی در مردان 54/7±58/24 و در زنان 42/9±96/27 می‌باشد. میانگین نمره حمایت اجتماعی در زنان بالاتر از مردان بود. حمایت اجتماعی با جنسیت(001/0P=)، تحصیلات (00/0P=)، شغل (001/0 P=)، رضایت از وضعیت اقتصادی(00/0P=)، داشتن بیماری مزمن (00/0P=) و وضعیت اقامت(041/0P=) از لحاظ آماری رابطه معنی‌دار نشان داده شد. اما بین حمایت اجتماعی و محل تولد رابطه آماری معنی داری مشاهده نشد (093/0P=). نتیجه‌گیری: با توجه به یافته‌های این مطالعه، عوامل فردی و محیطی مختلفی می‌توانند در حمایت اجتماعی مهاجرین تأثیرگذار باشند؛ همچنین وضعیت حمایت اجتماعی در مهاجران افغان متوسط می‌باشد؛ لذا نیاز به تأمین حمایت اطلاعاتی، عاطفی و ابزاری برای بالا بردن وضعیت حمایت اجتماعی در آن‌ها محسوس می‌باشد.

Title: خوش بینی علمی معلمان و رفتار شهروندی سازمانی

http://hsr.mui.ac.ir/index.php/jhsr/article/view/1199

مقدمه: خوش بینی علمی در مورد معلمان، یک سازۀ جدید است. پژوهش حاضر با هدف بررسی خوش بینی علمی معلمان مقطع ابتدایی شهر شیراز و رفتار شهروندی سازمانی آنها انجام شده است. روش ها: روش تحقیق توصیفی از نوع همبستگی است. جامعه آماری این پژوهش، تمام معلمان مرد و زن شاغل به کار در سال تحصیلی 1392-1391 مدارس دولتی شهر شیراز در مقطع ابتدایی بودند که با استفاده از روش نمونه گیری خوشه ای چند مرحله ای، 200 نفر از آنان انتخاب شدند. در مجموع حجم نمونه این پژوهش به 124 نفر کاهش یافت. در این تحقیق از شش پرسشنامه استفاده شد که شامل خوش بینی علمی (اعتماد، خود کارآمدی و تأکید علمی)؛ رفتار شهروندی سازمانی؛ گرایشات خوش بینانه؛ باورها و فعالیت های تدریس دانش آموز محور می شد. یافته ها: نتایج تحقیق نشان داد که میان خوش بینی علمی ، رفتار شهروندی، گرایشات خوش بینانه و فعالیت های دانش آموز محور معلمان رابطه مثبت و معنی دار برقرار است. تفاوت معنی داری در خوش بینی معلمان مرد و زن وجود ندارد و میزان خوش بینی آنها از حد متوسط بالاتر است. نتیجه گیری: با افزایش گرایشات خوش بینانه معلمان نسبت به زندگی، خوش بینی علمی آنها افزایش می یابد. با افزایش فعالیت های دانش آموز محور معلمان ، خوش بینی علمی آنها افزایش می یابد. خوش بینی علمی معلمان قادر به پیش بینی رفتار شهروندی سازمانی آنها است. نتایج تحلیل مسیر  نمایانگر این بود که مدل مورد نظر با داده ها برازش کامل دارد.

Title: بررسی کیفی وجود پنج آنتی بیوتیک در یک تصفیه خانه فاضلاب شهری واقع در منطقه مرکزی ایران

http://hsr.mui.ac.ir/index.php/jhsr/article/view/1233

مقدمه: هدف این مطالعه بررسی وجود پنج آنتی بیوتیک متداول انسانی و حیوانی شامل آمپی سیلین، اکسی تتراسیکلین، انروفلوکساسین، تایلوزن و سیپروفلوکساین بر اساس تکنولوژی استخراج فاز جامد-کروماتوگرافی مایع-اسپکترومتری جرمی دوگانه (SPE-LC-MS/MS) در یک تصفیه خانه فاضلاب در بخش مرکزی ایران بود. روش ها: در بهمن و اسفند ماه سال 1390، به روش نمونه برداری های گرب و غیرفعال، 4 نمونه از ورودی و خروجی تصفیه خانه فاضلاب مورد نظر برداشت شدند. نمونه های مذکور با استفاده از روش استخراج فاز جامد (SPE) آماده شدند و مواد استخراج شده توسط دستگاه کروماتوگرافی مایع اسپکترومری جرمی با یونیزاسیون الکترواسپری در مُد مثبت آنالیز شدند. یافته ها: نتایج نشان داد که انروفلوکساسین، اکسی تتراسیکلین و تایلوزین در هیچ یک از نمونه ها یافت نشدند. با این حال، آمپی سیلین و سیپروفلوکساسین در هر دو نمونه های گرب و غیرفعال برداشت شده از فاضلاب ورودی به تصفیه خانه و پساب خروجی از تصفیه خانه شناسایی گردیدند. نتیجه گیری: بدلیل برخی محدودیت های فنی و اقتصادی و فقدان داده های بازیابی[1] و اثر ماتریکس[2] معتبر، حضور آنتی بیوتیک های مورد بررسی بصورت کیفی گزارش گردید. نتایج نشان داد که نمونه برداری غیرفعال نتایج مشابهی با روش نمونه برداری گرب در شناسایی آنتی بیوتیک ها در فاضلاب خام ورودی و پساب خروجی از تصفیه خانه فاضلاب دارد. حضور آمپی سیلین و سیپروفلوکساسین در فاضلاب خام ورودی و پساب خروجی از تصفیه خانه فاضلاب مورد نظر ممکن است منجر به ظهور بالقوه باکتری های مقاوم شود که می بایست در تحقیقات آتی مدنظر قرار گیرد. [1] Recovery [2] Matrix Effect

Title: بررسي کميت و کيفيت شيرابه تولیدی از مراحل مختلف فرايند توليد کمپوست زباله شهري به روش ويندرو

http://hsr.mui.ac.ir/index.php/jhsr/article/view/1473

مقدمه:يكي از مهمترين مشكلات جانبي توليدكمپوست دركشور ما، وجود مقادير زياد رطوبت در زباله هاي شهري است كه منجر به توليد حجم زياد شيرابه مي گردد. هدف از انجام این مطالعه، بررسي کميت و کيفيت شيرابه تولیدی در مراحل مختلف فرايند توليد کمپوست زباله شهري به روش ويندرو می باشد. روش ها: اين مطالعه از نوع توصیفی بوده و عمل نمونه‌برداري به منظور تعیین کیفیت شیمیایی شیرابه ناشی از مراحل مختلف پردازش کمپوست، در 4 فصل سال و بطور ماهیانه انجام ‌شد. پارامترهای مورد آزمایش شاملpH،COD ،TSS ،EC،آمونياك،فسفر و فلزات سنگین(Cd,Zn,Pb,Cr) بود. نمونه برداری از  مراحل سالن دريافت، خرد کن و سايت تجزيه انجام شد. نتایج:متوسط کل دبی شیرابه خروجي از کارخانه کمپوست 4/0بوده که حداکثر    2/1 و حداقل   2/0 اندازه گیری شد. دبی خروجی از سالن دریافت حدود 04/0 ، از قسمت خردکن زباله و پرس زباله   08/0 و دبی شیرابه سایت تجزیه با   3/0 (حدود 70% کل دبی خروجی) بیشترین مقدار بوده است. میانگین غلظت COD در سالن دریافت زباله، خردکن ، پرس، سایت تجزیه و حوضچه تبخیری به ترتیب 39864، 104643، 29898، 119592 و 99660  میلی گرم در لیتر بوده است. بحث و نتیجه گیری: بیشترین دبی و آلودگی آلی شیرابه از سایت تجزیه و کمترین آن از بخش خرد کن تولید می شود. بیشترین تولید غلظت فلزات سنگین در این بخش است که به دلیل ورود فلزات به شیرابه طی عملیات خرد کردن می باشد. تفکیک از مبدا و بازیافت زباله مهمترین راه حل کاهش تولید شیرابه و آلودگی آن می باشد.

Title: بررسی علل تناقضات آماری ازدیدگاه مدیران ومسئولان آمارمراکزوابسته به دانشگاه علوم پزشکی اصفهان

http://hsr.mui.ac.ir/index.php/jhsr/article/view/1639

مقدمه: وجودآمار و اطلاعات صحیح در هر سازمانی نقش تعیین کننده ای داشته و در تجزیه و تحلیل عملکرد آن سازمان بسیار مهم است. مدیران از عمده ترین گروههایی هستند که از نتایج فعالیتهای آماری استفاده می کنند و مسئولان آمار موظف به جمع آوری و آماده کردن، تحلیل و تفسیر اطلاعات هستند. هدف از این پژوهش، بررسی علل تناقضات آماری از دیدگاه مدیران و مسئولان آمار مراکز وابسته به دانشگاه علوم پزشکی اصفهان بود. روش ها:این مطالعه، توصیفی- مقطعی با مشارکت 113نفر از مدیران و مسئولان آمار مراکز وابسته به دانشگاه علوم پزشکی اصفهان که با روش سرشماری نمونه گیری شدند انجام شد. گردآوری داده ها با پرسش نامه ای محقق ساخته، در قالب 6 دسته علل تناقضات آماری انجام گرفت.  داده ها با استفاده از روشهای آمار توصیفی و آزمون آنالیز واریانس و با نرم افزار SPSS تحلیل گردید. یافته ها: بیشترین میانگین نمره درکل گروههای مورد بررسی به علل تناقضات آماری مرتبط با قوانین و دستورالعمل ها(63/0±95/3)، هماهنگی (71 /0±90/3)، مدیریت (63/0±89/3) و کنترل و نظارت (72/0±87/3) اختصاص داده شد که مهمترین علل تناقضات آماری بودند. میانگین نمره علل تناقضات آماری مرتبط با قوانین ودستورالعمل ها (003/0P =)، هماهنگی(042/0 P =) و عوامل فرآیندی تولید و ارائه آمار (P <0/001) بین گروههای مورد بررسی تفاوت معنی دار دارد. نتیجه گیری: با توجه به این که مهمترین علل تناقضات آماری در دانشگاه علوم پزشکی اصفهان از دید مسئولان آمار در مقام تولیدکنندگان و مدیران درمقام مصرف کنندگان آمار، علل مرتبط با قوانین و دستورالعمل ها، هماهنگی، مدیریت و کنترل و نظارت می باشد، باید برنامه ریزی های مدونی به منظور به حداقل رساندن و حذف تناقضات نمود.

Title: بررسی فریضه نماز در دانشجویان جدید الورود دانشگاه علوم پزشکی اصفهان در سال 1391

http://hsr.mui.ac.ir/index.php/jhsr/article/view/1172

مقدمه: با توجه به اهمیت فریضه نماز به عنوان یکی از مهم‌ترین شاخصهای عملی دین داری؛ این مطالعه به بررسی فریضه نماز در میان دانشجویان پرداخته است. روش‌ها: این مطالعه توصیفی تحلیلی در دانشگاه علوم پزشکی اصفهان انجام شد و کل دانشجویان جديدالورود نیمسال اول تحصیلی 1391 در تمام رشته‌ها به صورت سرشماری مورد بررسی قرار گرفتند. معیار ورود مسلمان بودن و تمایل به شرکت در مطالعه و معیار خروج عدم تمایل به شرکت در مطالعه بود. اطلاعات با پرسش‌نامه محقق ساخته به روش خود ایفاء توسط دانشجویان داوطلب جمع‌آوری شد. اطلاعات با نرم افزار SPSS نسخه 18 با آمار توصيفي و استنباطي تجزیه و تحلیل گردید. یافته‌ها: 70٪ از دانشجویان در این مطالعه شرکت نمودند از این تعداد  7/74% دختر، 2/90% مجرد بودند. در بعد احساسی عاطفی (دارا بودن نظر مثبت و احساس خوشایند) 7/2% از دانشجویان ضعیف و 3/26% متوسط و 71% مطلوب بودند. در بعد رفتاری4/57% تقریبا همیشه نماز می‌خواندند و 4/1% از دانشجویان هیچگاه نماز نمی‌خواندند، 7/18% از دانشجویان بیشتر وقت‌ها و 5/7% همیشه در نماز جماعت شرکت می‌کردند، تنها 3/10% هیچ وقت در نماز جماعت شرکت نمی‌کردند، 1/38% از دانشجویان بیشتر وقت‌ها و 8/19% تقریبا همیشه در اول وقت نماز می‌خواندند. نتیجه‌گیری: با توجه به اهمیت و ظرافت موضوع؛ تدوین برنامه آموزشی موثر در راستای تقویت این فریضه به ویژه برای دانشجویان ساکن خوابگاه پیشنهاد می‌گردد.

Title: نقش توكل در تأمين سلامت رواني انسان

http://hsr.mui.ac.ir/index.php/jhsr/article/view/1030

مقدمه: يكي از معضلات عصر كنوني، اضطراب، نااميدي و احساس پوچ‌گرايي در ميان انسان‌ها است كه زمينه‌ساز مشكلات بسياري در زندگي بشر خواهد بود تا جايي كه فشار رواني را بيماري تمدن جديد نام نهاده‌اند. همين امر اهميت پرداختن به اين مسأله و پيداكردن راهي براي درمان وپيشگيري از به ‌خطر افتادن سلامت روان انسان‌ها را دوچندان خواهد كرد. روش‌ها: در اين مطالعه كه با روش كتابخانه‌اي صورت گرفته است، توكل به‌عنوان يكي از راه‌هاي پيشگيري و درمان فشارهاي رواني كه حتي مورد توجه روانشناسان غربي نيز قرار گرفته، مطرح گرديده است. یافته‌ها: توكل به‌ معناي تكيه كردن بر خداوند و واگذاري تمام امور به او به همراه تلاش و در پايان حس رضايتمندي است كه توكل واقعي مي‌تواند زمينه‌ساز اميد، حسن‌ظن، صبر، دوري از صفات رذيله انساني، شوق در راه نيل به اهداف، هدفمند ديدن دنيا و اتفاقات آن و در نهايت بهبود كيفيت زندگي و تأمين سلامت رواني انسان‌ها گردد و آيات و روايات متعددي نيز مؤيد اين مطلب است. نتیجه‌گیری: انسانهاي متوكل به واسطه توكل و اعتمادشان بر خداوند متعال، انسان‌هايي اميدوار، صبور و داراي حسن‌ظن به انسان‌هاي ديگر و اتفاقات پيرامونشان هستند زيرا دنيا و مشكلاتش را هدفمند و هرآنچه را كه اتفاق مي‌افتد جز خير و مصلحت خويش نمي‌پندارند، بنابراين نسبت به ديگران از آرامش رواني بيشتري برخوردارند.

Title: تاثير رويکرد زوج درماني کوتاه مدت راه حل– محور بر سازگاري زناشويي در زنان شهر قم

http://hsr.mui.ac.ir/index.php/jhsr/article/view/1039

مقدمه: سازگاري زناشويي يکي از گسترده‌ترين مفاهيم براي تعيين و نشان دادن ميزان شادي و ميزان پايداري رابطه زن و شوهر است. سازگاري زناشويي بر بسياري از ابعاد زندگي فردي و اجتماعي انسان‌ها تاثير مي‌گذارد. هدف ‏پژوهش حاضر بررسي تاثیر رويکرد راه‏حل-محور بر افزايش سازگاري‏زناشويي زنان شهر قم در سال 91-92صورت گرفته است. روش‌ها: اين پژوهش نيمه‏تجربي است و در آن از پيش‏آزمون و پس‏آزمون با گروه کنترل استفاده شده است. جامعه پژوهش شامل آن دسته از زوجين مراجعه‏کننده به مرکز مشاوره خانواده مي‏باشد که به صورت خود ‏معرف مراجعه کرده بودند. نمونه پژوهش شامل 60 نفر از زنانی است که داوطلب به شرکت در مطالعه بودند، به صورت نمونه در دسترس و تصادفي در گروه آزمايش و کنترل قرار گرفتند. ابزار پژوهش پرسش‏نامه دموگرافیک و پرسش‏نامه سازگاري‏ زناشويي اسپاينر بود. براي تجزيه و تحليل داده‏ها از نرم افزار SPSS نسخه 16با استفاده از روش‏هاي آمار توصيفي و تحليلی (کوواريانس) استفاده شد. یافته‌ها: نتايج نشان داد كه آموزش گروهي براساس نظريه راه حل محور بر سازگاري زناشويي تاثير معنی‌دار داشته است (05/0P<). همچنین در ابعاد رضایت، همبستگی نیز و ابراز محبت تاثير معنی‌دار داشته است (05/0P<). نتیجه‌گیری: لذا با توجه به بالا رفتن نرخ طلاق و کاهش سازگاری و رضایت زناشویی در دو دهه گذشته در كشور، درمان راه حل – محور با کمک به زوجین براي توسعه راه حل‌هاي مؤثر و پیدا کردن موارد استثنا در زندگی می‌تواند سازگاری زناشویی را افزایش و از طلاق جلوگیری کرد.

Title: بررسی رضایت شغلی ماماهای شاغل در بخش زایشگاه بیمارستان‌های شهر اصفهان

http://hsr.mui.ac.ir/index.php/jhsr/article/view/874

مقدمه: از آنجا که ماماها نقش مهمی را در سلامت خانواده و جامعه ایفا می‌کنند و افزایش رضایت شغلی آنها ، باعث افزایش کیفیت خدمات مراقبتی و درمانی ارایه شده به دو قشر حساس جامعه یعنی مادران و نوزادان و در نتیجه رضایت و سلامت خانواده‌ها خواهد شد، توجه به سلامت و رضایت شغلی آنها از اهمیت زیادی برخوردار است، بنابراین این مطالعه با هدف بررسی میزان رضایت شغلی ماماهای شاغل در بخش زایشگاه انجام شد. روش‌ها: این مطالعه توصیفی تحلیلی از نوع مقطعی می‌باشد. جامعه پژوهش شامل 106 مامای شاغل در زایشگاه‌های شهر اصفهان بود که به شکل تصادفی انتخاب و وارد مطالعه شدند. روش جمع‌آوری اطلاعات، پرسش‌نامه‌ رضایت شغلی (JDI) می‌باشد (شامل 55 سؤال) که توسط اسمیت، کندال و هالین تدوین شده و روایی و پایایی آن در مطالعات قبلی تأیید شده است.داده‌ها با استفاده از نرم‌افزار SPSS نسخه 18 و آزمون‌های آماری توصیفی و استنباطی (همبستگی پیرسون، همبستگی اسپیرمن، آزمون آنالیز واریانس یکطرفه و آزمون t مستقل) مورد تجزیه و تحلیل قرار گرفتند. سطح معنی‌داری کمتر از 05/0 در نظر گرفته شد. یافته‌ها: نتایج نشان داد که میانگین نمره رضایت شغلی ماماها 43/16± 75/44 (از 100) می‌باشدکه 8/23% ماماها دارای رضایت شغلی کم و 5/69% دارای رضایت شغلی متوسط و 7/6% دارای رضایت شغلی زیاد بودند. بیشترین میزان رضایت در بعد رضایت از سرپرست (9/61%) و بیشترین نارضایتی از میزان حقوق و مزایا (8/83%) و فرصت‌های ترفیع شغلی (1/57%) بوده است. همچنین بین رضایت کلی شغلی با تمام ابعاد آن ارتباط و همبستگی مثبت و معنی‌دار آماری دیده شد. در این مطالعه بین سطح رضایت کلی شغلی با سن، میزان سابقه کاری، میزان تحصیلات و وضعیت تأهل افراد رابطه معنی‌دار مشاهده نشد. در حالی که بین سطح رضایت کلی شغلی با میزان حقوق و مزایا و وضعیت استخدامی ارتباط آماری معنی‌دار دیده شد (05/0 P<). نتیجه‌گیری: نتایج این مطالعه نشان داد که فقط درصد کمی از ماماهای شاغل در بخش زایشگاه دارای رضایت شغلی بالایی هستند، بنابراین پیشنهاد می‌شود که مسؤولین محترم با بکارگیری تدابیری همچون بهبود شرایط محیط کار، افزایش حقوق و مزایا، ایجاد فرصت‌های ترفیع شغلی و همچنین قوانین حمایتی موجود، سبب افزایش رضایت شغلی و ایجاد یک محیط کاری بهینه و مطلوب جهت ماماها و در نتیجه یک محیط رضایت بخش برای بیماران و مراجعین گردند.
